# Annual Meeting of the Erie County Medical Society

**Published:** 1884-03

**Authors:** Arthur M. Barker

**Affiliations:** Secretary


### Society Reports.

ANNUAL MEETING OF THE ERIE COUNTY MEDICAL SOCIETY.

The annual meeting of the Erie County Medical Society was held January 8, 1884, in the amphitheatre of the College, corner of Main and Virginia streets. The attendance was unusually large, among those present being Drs. Nott, Briggs, F. H. Potter, W. W. Potter, Thomas Lothrop, G. E. Fell, Harrington, John Cronyn, J. L. C. Cronyn, Charles Ring, William Ring, Ingraham, Wyckoff, Warren, Mann, Edward Storck, Howe Hopkins, Hubbell, Daniels, Peterson, Samo, Gay, Dorland, Mary Runner, Wall, Lapp, Rochester, Stockton, Barker, Eli Long, B. G. Long, Gregory, Peterson, King, Mynter, Tremaine, Hauenstein, Thornton, Lynde, J. C. Greene, Dagenais, Barnes, C. L. Dayton, Dambach, Armstrong, Pryor, Macneil, Snow, Hoyer, Frank, Heath, Keene, Putnam, Granger, Mary Berkes, Andrews, Jewett, Montgomery, Phelps, Fredrich, Abbott, Johnson, J. S. Smith, Vaughn, Clark, Coakley, O'Brien, Ballou, A. G. Gumar, Crawford, Fowler, Meisberger, Bartlett, Abbott of Hamburg; Brecht, Boardman, L. P. Dayton, Davidson, Pettit, Van Peyma, William H. Jackson, B. H. Grove, Charles Cary, Walsh.

In the absence of President E. S. H. Nott, and also of the Vice-President, Dr. Ring was appointed chairman *pro tem.*

The Secretary, Dr. Barker, read the minutes of the previous meeting, which were approved. The minutes of the special meeting held since the semi-annual meeting were also approved without reading.

The reception of reports from the various committees was the next business. Dr. E. T. Dorland, as chairman of the Committee on Membership, reported that they had examined the credentials of the following candidates for membership and found them duly qualified and registered, and therefore recommended their admission as members of the Erie County Medical Society: Prof. R. A. Witthaus, William Meisburger, W. A. D. Montgomery, B. G. Long, Carleton R. Jewett, John D. Niemand,

F. W. Sweetland, W. H. Thornton, A. G. Gumar, and Mary Berkes.

On suggestion of Dr. Barker, the newly elected members were introduced to the society by Dr. Dorland.

The following applications for membership were received: Dr. William B. Hawkins, Dr. Roswell R. Park, Dr. Alpheus Prince, Dr. Herman Bauer, Dr. Albert E. Persons, Dr. Herbert Mickle, Dr. Julius H. Potter, Dr. F. R. Campbell, Dr. Andrew F. Hellwig, Dr. Reuben M. Root. They were referred until the next meeting.

The Secretary submitted a report on the by-laws of the society which on motion of Dr. Howe was received. The names of the present members to be added, and 300 copies ordered printed at an expense not exceeding $30.

At this part of the proceedings Dr. Nott, the President, entered the room and took the Chair.

The report of the Treasurer, Dr. F. W. Abbott, was next presented, and showed a balance of $8.30 in treasury. On motion of Dr. Howe the report was received and referred to an auditing committee consisting of Drs. Wyckoff, Gay and Howe, appointed by the President.

Dr. J. B. Samo, Librarian, presented his report, which was accepted without discussion.

The Board of Censors, through their chairman, Dr. Edward Storck, submitted a report with reference to the action of the board as to a bill presented to the last Legislature to legalize certain institutions in this State that had no legal charter and to confer on them the right of granting diplomas. They had prepared a written protest to Governor Cleveland, which was duly presented and resulted in the Governor refusing to sign the bill, which therefore failed to become a law. The report further pointed out the legal conditions under which medical schools had the power to grant diplomas, one of the conditions being that such school or college must be organized under the law of 1870, by which the deposit of a bond for $50,000 with the State Comptroller is required. Reference was also made to the prosecution of a man named Leo Labolsky, who claimed to hold a certificate from Dr. Brayton, dean of the faculty of the "College of Physicians and Surgeons," but who left town, forfeiting his bail of $100 under which he was placed by Police Justice King. In conclusion the report referred to many cases of violation of the fee bill and medical ethics and of unprofessional conduct on the part of some members of the society. No action had been taken so far, but the board suggested that either the board of this year or a special committee be authorized to investigate these cases, and prefer charges against the offenders. On one of these violations the board had taken action and asked the society to give immediate consideration to the following resolution:

WHEREAS, Members of the medical profession as members of mutual aid societies, secret orders or other benevolent associations, are asked to make examinations of applicants as thorough and complete as for any life insurance company for the amount of one dollar, therefore,

*Resolved*, That it shall be considered dishonorable and shall be cause for expulsion from the society for any member to make or cause to be made a medical examination, as asked by the certificates, for any such societies or individual for a less fee than $3.

Considerable discussion ensued on the resolution, many of the members considering the wording of it as too severe, while others thought that the present mode of examination by benefit societies was a sham, and that better medical examinations and fewer assessments would be more beneficial to the societies in question. The report was finally received and the resolution laid on the table.

Dr. H. R. Hopkins, of the Committee on Legislation, reported progress. The interest in the matter, he said, was most encouraging. From every county in the State words of commendation for the project had been received. In some counties action had been taken by societies endorsing the bill. The Erie County Society was to be congratulated upon the fact that this matter was now before the entire medical profession of the State and that there was concerted action in behalf of the object. The committee recommended for adoption a resolution urging the Senator and Assemblymen of this district to interest themselves in behalf of the bill for the establishment of a State Board of Examiners, and secure its passage.

The following is the resolution of the Committee on Legislation:

*Resolved*, That the Medical Society of the county of Erie urge upon the Senator from the Thirty-first District, the Hon. Robert C. Titus, and the members of Assembly from the county of Erie, Hon. Cornelius Donahue, Hon. Frank Sipp, Hon. Geo. Clinton, Hon. T. W. Jackson and Hon. David J. Wilcox, to use all laudable effort in the present session of the Legislature to secure the passage of the act proposed for the purpose of establishing the Medical Faculty or University of the State of New York.

*Resolved*, That copies of the resolutions be transmitted to the members of the Legislature above mentioned, and also to the secular and medical press.

The report was accepted and the resolution adopted.

The following officers were elected for the ensuing year:

President—Dr. J. C. Greene.

Vice-President—Dr. Judson B. Andrews.

Secretary—Dr. Edward Clark.

Treasurer—Dr. F. W. Abbott (re-elected).

Librarian—Dr. J. B. Samo (re-elected).

Board of Censors—Dr. E. Storck, Dr. H. R. Hopkins, Dr. A. H. Briggs, Dr. P. W. Van Peyma, Dr. F. F. Hoyer.

Essayist—Dr. P. W. Van Peyma; Dr. Herman Hartwig, alternate.

Committee on Membership—Dr. E. T. Dorand, Dr. Herman Mynter, Dr. T. M. Johnson.

Dr. Barker declined re-election as Secretary.

Dr. John Hauenstein tendered his resignation as member of the Legislative Committee. Dr. J. C. Greene was elected to fill the vacancy.

A vote of thanks was tendered the retiring President, Secretary and Treasurer for their services during the past year.

Dr. Nott, by request, withheld his annual address until the June meeting.

At one o'clock the meeting adjourned *sine die*.

Orders on the Treasury were directed to be drawn for the following: Peter Paul & Bro., $13.90; John Ferguson, janitor, $10; State society dues, $25; Sydenham society, ——; insurance, ——.

ARTHUR M. BARKER, M. D.,
*Secretary.*

